# Slim Tree-Cut Width

**DOI:** 10.1007/s00453-024-01241-4

**Published:** 2024-06-01

**Authors:** Robert Ganian, Viktoriia Korchemna

**Affiliations:** Algorithms and Complexity Group, TU Wien, Vienna, Austria

**Keywords:** Tree-cut width, Structural parameters, Graph immersions

## Abstract

Tree-cut width is a parameter that has been introduced as an attempt to obtain an analogue of treewidth for edge cuts. Unfortunately, in spite of its desirable structural properties, it turned out that tree-cut width falls short as an edge-cut based alternative to treewidth in algorithmic aspects. This has led to the very recent introduction of a simple edge-based parameter called edge-cut width [WG 2022], which has precisely the algorithmic applications one would expect from an analogue of treewidth for edge cuts, but does not have the desired structural properties. In this paper, we study a variant of tree-cut width obtained by changing the threshold for so-called thin nodes in tree-cut decompositions from 2 to 1. We show that this “slim tree-cut width” satisfies all the requirements of an edge-cut based analogue of treewidth, both structural and algorithmic, while being less restrictive than edge-cut width. Our results also include an alternative characterization of slim tree-cut width via an easy-to-use spanning-tree decomposition akin to the one used for edge-cut width, a characterization of slim tree-cut width in terms of forbidden immersions as well as approximation algorithm for computing the parameter.

## Introduction

Understanding which structural properties of inputs allow us to overcome the inherent intractability of problems of interest is a fundamental research area in computer science. In the context of parameterized complexity, one typically approaches this by asking which structural parameters of the input (or its graph representation) give rise to a fixed-parameter algorithm for a targeted problem. Treewidth [36] is the most prominent example of such a structural parameter, and can be viewed as a guarantee that a graph is iteratively decomposable along small vertex separators. Many problems are known to be fixed-parameter tractable when parameterized by treewidth—and for those that are not, there is a well-studied hierarchy of more restrictive^1^ parameters based on vertex separators or vertex deletion that can sometimes be used instead (see, e.g., Figure 1 in [3]). Examples of such parameters include the vertex cover number [11, 14], the feedback vertex number [2, 27] and treedepth [19, 26, 32, 33].

However, such vertex based parameters seem ill suited for handling some problems. Consider, for instance, the classical Edge Disjoint Paths problem (EDP): unlike Vertex Disjoint Paths, EDP remains NP-hard not only on graphs of bounded treewidth, but even on graphs with a vertex cover number of at most 3 [13]. While this effectively rules out the use of all parameters based on vertex separators, there is an intuitive expectation that EDP should be fixed-parameter tractable w.r.t. parameters that can guarantee an iterative decomposition of the graph along small edge cuts. Indeed, EDP is known to be fixed-parameter tractable w.r.t. two basic parameterizations which provide such a guarantee: the feedback edge number [20] and treewidth plus maximum degree [21].

An ideal solution for handling such problems on more general inputs would be to use an alternative to treewidth that would be designed around edge cuts rather than vertex separators, one which would provide a unified justification for tractability w.r.t. the two basic “edge-cut restricting” parameterizations mentioned above. A candidate for such a parameter was proposed by Wollan, who defined *tree-cut width* along with *tree-cut decompositions* and described these as a variation of tree decompositions based on edge cuts instead of vertex separators [38]. But while it is true that “tree-cut decompositions share many of the natural properties of tree decompositions” [31], from the perspective of algorithmic design tree-cut width seems to behave differently than an edge-cut based alternative to treewidth. Indeed, not only does it fall short of yielding a fixed-parameter algorithm for EDP [20], it also fails to provide such algorithms for other problems one would expect to be fixed-parameter tractable w.r.t. an edge-cut based analogue to treewidth. In fact, out of twelve such problems where a tree-cut width parameterization has been pursued so far, only four are fixed-parameter tractable [16, 17] while eight turn out to be ${{{\textsf {W}}}} [1]$-hard [5, 16, 18, 20, 24] (see the Related Work at the end of the Introduction for details).

Very recently, Brand, Ceylan, Ganian, Hatschka and Korchemna [4] introduced a parameter called *edge-cut width* which aimed at filling this gap in our understanding of edge-cut based graph parameters. On the algorithmic side, edge-cut width has precisely the properties one could hope to see in an edge-based analogue to treewidth: not only does it yield fixed-parameter algorithms for all twelve “candidate” problems [4], but it is also based on a very simple type of decomposition that is much easier to use than tree-cut decompositions. That being said, already the authors of that paper noted that the structural properties of edge-cut width are far from ideal—for instance, it is the only algorithmically used parameter we are aware of that is not closed under vertex deletion. Moreover, while edge-cut width is less restrictive than the feedback edge number, unlike tree-cut width it is incomparable to treewidth plus maximum degree (even in an asymptotic sense). Because of this, it cannot act as a common generalization that would capture both of these basic approaches of enforcing decomposability along small edge cuts.

**Contribution.**    In this paper, we identify a graph parameter which combines the advantages of tree-cut width and edge-cut width while avoiding all of the shortcomings listed above. However, before we introduce it, it will be useful to establish at least some intuitive understanding of tree-cut width^2^.

A graph *G* has tree-cut width at most *k* if it admits a tree-cut decomposition *T* of width *k*, whereas *T* is a rooted tree and its nodes act as bags that form a partitioning of *V*(*G*). A non-root node *t* of *T* defines an edge cut between all vertices in the subtree rooted at *t*, and the rest of the graph. The definition of tree-cut width then restricts, for each node *t*, the number of its children defining an edge cut of size greater than 2. The constant “2” here arises from the structural properties Wollan aimed for when defining tree-cut width [38]; however, let us now pose the following question: How would the parameter change if we used a different constant *c* here instead?

On one hand, it is not difficult to observe that values of $c>2$ would immediately lead to parameters without the properties we are aiming for, since these would be constant for, e.g., all 3-regular graphs. On the other hand, we show that for $c=0$, one obtains an asymptotically equivalent characterization of one of the previously mentioned basic edge-cut restricting parameterizations: treewidth plus maximum degree. Our parameter of interest is then the outcome of setting $c=1$; since this can be viewed as a variant of tree-cut width where all but a few children of each node need to have “even slimmer” edge-cuts, we refer to it as *slim tree-cut width* (${\text {stcw}}$).

**Fig. 1:**
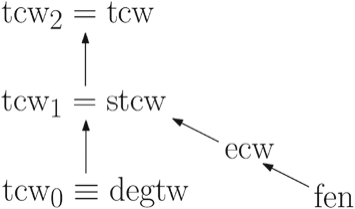
Hierarchy of graph parameters based on edge cuts. Here ${\text {ecw}}$ denotes edge-cut width and ${\text {degtw}}$ denotes treewidth plus maximum degree. ${\text {tcw}}_i$ denotes the parameter obtained from tree-cut width by setting the constant *c* described above to *i*. An arrow from *p* to *q* represents the fact that *p* is more restrictive than *q*, while asymptotic equivalence is depicted by $\equiv$

On the structural side, we show that ${\text {stcw}}$ inherits the desirable properties of its “non-slim” namesake. In particular, unlike edge-cut width [4], ${\text {stcw}}$ is closed under edge sums, vertex and edge deletion, as well as under the graph immersion operation. Similarly as Wollan did for tree-cut width [38], we also provide a set of forbidden immersions asymptotically characterizing ${\text {stcw}}$. Furthermore, we show that ${\text {stcw}}$ is a common generalization of edge-cut width (and hence the feedback edge number), and treewidth plus maximum degree (see Fig. 1).

Next, as one of our arguably most surprising results, we show that ${\text {stcw}}$ is asymptotically equivalent to a slight generalization of edge-cut width: instead of measuring the width over the input graph *G*, we ask for the minimum edge-cut width of any supergraph of *G*. The transformation between these parameters is constructive and has interesting algorithmic implications. First of all, when designing algorithms it allows us to avoid the use of often cumbersome tree-cut decompositions, and instead opt for the simpler decompositions used for edge-cut width—which are nothing else than spanning trees (in this case of a supergraph). Second, all of the fixed-parameter algorithms recently designed for edge-cut width [4] rely on a dynamic programming traversal of the spanning tree, and can be straightforwardly adapted to work on spanning trees of supergraphs instead. This means that one can essentially reuse the same proofs to establish fixed-parameter tractability of all considered “candidate” problems w.r.t. ${\text {stcw}}$.

Naturally, a crucial prerequisite for algorithmically applying ${\text {stcw}}$ is that we can actually compute it, or more precisely compute a suitable decomposition for graphs of small ${\text {stcw}}$. While the problem of computing an optimal decomposition remains open even for tree-cut width, a fixed-parameter approximation algorithm was obtained by Kim, Oum, Paul, Sau and Thilikos [28] and this suffices for the purposes of establishing fixed-parameter tractability. We obtain a similar outcome here and also provide a fixed-parameter approximation algorithm for ${\text {stcw}}$, albeit with a worse approximation factor than for tree-cut width.

**Related Work.**   Tree-cut width parameterizations were typically considered for problems which are not fixed-parameter tractable (FPT) w.r.t. treewidth, but are FPT w.r.t. feedback edge number and also FPT w.r.t. treewidth plus maximum degree. The twelve candidate problems where tree-cut width parameterizations have been considered are shown in Table 1.

**Table 1:** The twelve candidate problems and their complexity w.r.t. edge-cut based parameters, where ${\text {degtw}}$ denotes the maximum degree plus treewidth. Slim tree-cut width provides a unified explanation for why these problems are FPT w.r.t. both edge-cut width and ${\text {degtw}}$, and lifts these results to more general inputs

Problem	tree-cut width	edge-cut width	${\text {degtw}}$	${\text {stcw}}$
Capacitated Vertex Cover	FPT [16]	FPT	FPT	FPT
Capacitated Dominating Set	FPT [16]	FPT	FPT	FPT
Imbalance	FPT [16]	FPT	FPT	FPT
Bounded Degree Deletion	FPT [17]	FPT	FPT	FPT
Edge Disjoint Paths	${{{\textsf {W}}}} [1]$-hard [20]	FPT [4]	FPT [21]	FPT
List Coloring	${{{\textsf {W}}}} [1]$-hard [16]	FPT [4]	FPT [16]	FPT
Precoloring Extension	${{{\textsf {W}}}} [1]$-hard [16]	FPT [4]	FPT [16]	FPT
Boolean Constraint Satisfaction	${{{\textsf {W}}}} [1]$-hard [16]	FPT [4]	FPT [37]	FPT
Bayesian Network Structure Learning	${{{\textsf {W}}}} [1]$-hard [18]	FPT [4, 18]	FPT [34]	FPT
Polytree Learning	${{{\textsf {W}}}} [1]$-hard [18]	FPT [4, 18]	FPT [18]	FPT
Min. Changeover Cost Arborescence	${{{\textsf {W}}}} [1]$-hard [24]	FPT [4]	FPT [25]	FPT
MSRTIL^a^	${{{\textsf {W}}}} [1]$-hard [5]	FPT [4]	FPT [1, 5]	FPT

^a^Maximum Stable Roommates with Ties and Incomplete Lists. For completeness, we note that the authors who showed ${{{\textsf {W}}}} [1]$-hardness w.r.t. tree-cut width also identified two additional restrictions which, when combined with tree-cut width, suffice for fixed-parameter tractability [5]

The structural properties of tree-cut width have also been studied in a number of recent papers [22, 23]. Last but not least, we note that a preprint exploring a different parameter that is aimed at providing an edge-based alternative to treewidth was recently authored by Magne, Paul, Sharma and Thilikos [30]; the parameter is based on different ideas and is incomparable to both tree-cut width and slim tree-cut width.

## Preliminaries

We use standard terminology for graph theory [9] and assume basic familiarity with the parameterized complexity paradigm including, in particular, the notions of *fixed-parameter tractability* and ${{{\textsf {W}}}} [1]$-*hardness* [8, 10]. Let $\mathbb {N}$ denote the set of natural numbers including zero. We use [*i*] to denote the set $\{0,1,\dots ,i\}$.

The *(open) neighborhood* of a vertex $x \in V(G)$ is the set $\{y\in V(G)~|~xy\in E(G)\}$ and is denoted by $N_G(x)$. For a vertex subset *X*, the neighborhood of *X* is defined as $\bigcup _{x\in X} N_G(x) \setminus X$ and denoted by $N_G(X)$; we drop the subscript if the graph is clear from the context. If *H* is a subgraph of *G*, we denote it by $H\subseteq G$. *Contracting* an edge $\{a,b\}$ is the operation of replacing vertices *a*, *b* by a new vertex whose neighborhood is $(N(a)\cup N(b)){\setminus } \{a,b\}$. For a vertex set *A* (or edge set *B*), we use $G-A$ ($G-B$) to denote the graph obtained from *G* by deleting all vertices in *A* (edges in *B*), and we use *G*[*A*] to denote the *subgraph induced on* *A*, i.e., $G- (V(G)\setminus A)$.

Let *G* be a graph and let *x*, *y* and *z* be three distinct vertices of *G* such that $(x,y),(y,z) \in E(G)$. To *lift* the pair of edges (*x*, *y*), (*y*, *z*) means to delete the edges (*x*, *y*) and (*y*, *z*) from *G* and add (if it doesn’t exist yet) a new edge (*x*, *z*). We say that *G* contains *H* as a *weak immersion* (denoted $H\le _I G$) if and only if *H* can be obtained from *G* by a sequence of edge deletion, vertex deletion, and lifting operations.

For a natural number *k*, we say that a graph *G* is a *k*-*edge sum* of vertex-disjoint graphs $G_1$ and $G_2$ if there exist vertices $v_i \in V(G_i)$ of degree *k* for $i = 1,2$ and a bijection $\pi : N_{G_1}(v_1) \rightarrow N_{G_2}(v_2)$ such that *G* is obtained from $(G_1 - \{v_1\}) \cup (G_2 - \{v_2\})$ by adding an edge $(v,\pi (v))$ for every $v\in N_{G_1}(v_1)$. In this case we write $G=G_1\oplus _k G_2$. Observe that the same pair of graphs may produce different *k*-edge sums.

Given two graph parameters $\alpha ,\beta : G\mapsto \mathbb {N}$, we say that $\alpha$ *dominates* $\beta$ if there exists a function *p* such that for each graph *G*, $\alpha (G)\le p(\beta (G))$. If $\alpha$ dominates $\beta$ but $\beta$ does not dominate $\alpha$, we often say that $\beta$ is more restrictive than $\alpha$; as an example, treewidth dominates the vertex cover number. Two parameters that dominate each other are called asymptotically equivalent.

**Tree-cut Width.**   The notion of tree-cut decompositions was introduced by Wollan [38], see also subsequent work by Marx and Wollan [31]. A family of subsets $X_1, \ldots , X_{k}$ of *X* is a *near-partition* of *X* if they are pairwise disjoint and $\bigcup _{i=1}^{k} X_i=X$, allowing the possibility of $X_i=\emptyset$.

### Definition 1

A *tree-cut decomposition* of *G* is a pair $(T,\mathcal {X})$ which consists of a rooted tree *T* and a near-partition $\mathcal {X}=\{X_t\subseteq V(G)~|~t\in V(T)\}$ of *V*(*G*). A set in the family $\mathcal {X}$ is called a *bag* of the tree-cut decomposition.

For any node *t* of *T* other than the root *r*, let $e(t)=ut$ be the unique edge incident to *t* on the path to *r*. Let $T_u$ and $T_t$ be the two connected components in $T-e(t)$ which contain *u* and *t*, respectively. Note that $(\bigcup _{q\in T_u} X_q, \bigcup _{q\in T_t} X_q)$ is a near-partition of *V*(*G*), and we use $E_t$ to denote the set of edges with one endpoint in each part. We define the *adhesion* of *t* (${\text {adh}}(t)$) as $|E_t|$; we explicitly set ${\text {adh}}(r)=0$ and $E(r)=\emptyset$. The adhesion of $(T,\mathcal {X})$ is then ${\text {adh}}(T, \mathcal {X})=\max _{t\in V(T)}{\text {adh}}(t)$.

The *torso* of a tree-cut decomposition $(T,\mathcal {X})$ at a node *t*, written as $H_t$, is the graph obtained from *G* as follows. If *T* consists of a single node *t*, then the torso of $(T,\mathcal {X})$ at *t* is *G*. Otherwise, let $T_1, \ldots , T_{\ell }$ be the connected components of $T-t$. For each $i=1,\ldots , \ell$, the vertex set $Z_i\subseteq V(G)$ is defined as the set $\bigcup _{b\in V(T_i)}X_b$. The torso $H_t$ at *t* is obtained from *G* by *consolidating* each vertex set $Z_i$ into a single vertex $z_i$ (this is also called *shrinking* in the literature). Here, the operation of consolidating a vertex set *Z* into *z* is to substitute *Z* by *z* in *G*, and for each edge *e* between *Z* and $v\in V(G)\setminus Z$, adding an edge *zv* in the new graph. We note that this may create parallel edges.

The operation of *suppressing* (also called *dissolving* in the literature) a vertex *v* of degree at most 2 consists of deleting *v*, and when the degree is two, adding an edge between the neighbors of *v*. Given a connected graph *G* and $X\subseteq V(G)$, let the *3-center* of (*G*, *X*) be the unique graph obtained from *G* by exhaustively suppressing vertices in $V(G) {\setminus } X$ of degree at most two. Finally, for a node *t* of *T*, we denote by $\tilde{H}_t$ the 3-center of $(H_t,X_t)$, where $H_t$ is the torso of $(T,\mathcal {X})$ at *t*. Let the *torso-size* ${\text {tor}}(t)$ denote $|\tilde{H}_t|$.

### Definition 2

The width of a tree-cut decomposition $(T,\mathcal {X})$ of *G* is defined as $\max _{t\in V(T)}\{ {\text {adh}}(t),$ ${\text {tor}}(t) \}$. The tree-cut width of *G*, or ${\text {tcw}}(G)$ in short, is the minimum width of $(T,\mathcal {X})$ over all tree-cut decompositions $(T,\mathcal {X})$ of *G*.

Without loss of generality, we shall assume that $X_r=\emptyset$. We conclude this subsection with some notation related to tree-cut decompositions. Given a tree node *t*, let $T_t$ be the subtree of *T* rooted at *t*. Let $Y_t=\bigcup _{b\in V(T_t)} X_b$, and let $G_t$ denote the induced subgraph $G[Y_t]$. A node $t\ne r$ in a rooted tree-cut decomposition is *thin* if ${\text {adh}}(t)\le 2$ and *bold* otherwise.

A tree-cut decomposition $(T,\mathcal {X})$ is *nice* if it satisfies the following condition for every thin node $t\in V(T)$: $N(Y_t)\cap (\bigcup _{b\text { is a sibling of }t}Y_b)=\emptyset$. The intuition behind nice tree-cut decompositions is that we restrict the neighborhood of thin nodes in a way which facilitates dynamic programming. Every tree-cut decomposition of width *k* can be transformed into a nice tree-cut decomposition of the same width in cubic time [16]. Moreover, the resulting nice decomposition has the following property. For a node *t*, let $B_t=\{ b\text { is a child of }t~|~|N(Y_b)|\le 2\wedge N(Y_b)\subseteq X_t \}$ denote the set of thin children of *t* whose neighborhood is a subset of $X_t$, and let $A_t= \{a\text { is a child of }t~|~a\not \in B_t \}$ be the set of all other children of *t*. Then $|A_t|\le 2k+1$ for every node *t* [16].

We refer to previous work [16, 28, 31, 38] for a detailed comparison of tree-cut width to other parameters. Here, we mention only that tree-cut width is dominated by treewidth and dominates treewidth plus maximum degree, which we denote ${\text {degtw}}(G)$. It also dominates the feedback edge number (the size of a minimum feedback edge set), denoted ${\text {fen}}(G)$.

### Lemma 1

([16, 31, 38]) For every graph *G*, ${\text {tw}}(G)\le 2{\text {tcw}}(G)^2+3{\text {tcw}}(G)$ and ${\text {tcw}}(G)\le {\text {fen}}(G)+1$ and ${\text {tcw}}(G)\le 4{\text {degtw}}(G)^2$.

**Edge-Cut Width.**   The notion of edge-cut width was introduced by Brand at al. [4]. For a graph *G* and a maximal spanning forest *T* of *G*, let the *local feedback edge set* at $v\in V$ be

$$\begin{aligned} E_{{\text {loc}}}^{G,T}(v)=\{uw\in E(G)\setminus E(T)~|~\text {the path between { u} and { w} in { T} contains }v\}. \end{aligned}$$

### Definition 3

The edge-cut width of the pair (*G*, *T*) is ${\text {ecw}}(G,T)=1+\max _{v\in V} |E_{{\text {loc}}}^{G,T}(v)|$, and the edge-cut width of *G* (denoted ${\text {ecw}}(G))$ is the smallest edge-cut width among all possible maximal spanning forests *T* of *G*.

### Proposition 1

([4]) For every graph *G*, ${\text {tcw}}(G)\le {\text {ecw}}(G) \le {\text {fen}}(G)+1$.

**Fig. 2:**
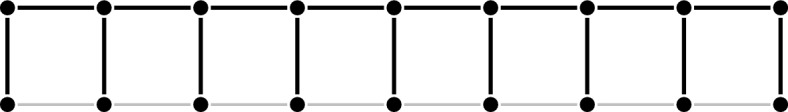
Example of a graph *G* with a spanning tree *T* (thick black) such that ${\text {ecw}}(G)={\text {ecw}}(G,T)=3$. The feedback edge number of *G* can be made arbitrarily large in this fashion

In fact, it was shown in [4] that the gaps in both inequalities can be arbitrary large, see Fig. 2 for a simple example of the second one.

Edge-cut width is not closed under vertex or edge deletions and is incomparable to ${\text {degtw}}$ [4]. However, the fact that its decomposition is simply a spanning tree makes it easier to work with in dynamic programming applications than, e.g., tree-cut decompositions [4].

## Refined Measures for Tree-Cut Decompositions

### Definitions and Comparison

Let us now define our parameter of interest, obtained by altering the threshold for when a vertex is suppressed (dissolved) in the definition of tree-cut width. Formally, let $(T,\mathcal {X})$ be some tree-cut decomposition of *G*. Given a connected graph *Q* and $X\subseteq V(Q)$, let the *2-center* of (*Q*, *X*) be the unique graph obtained from *Q* by exhaustively deleting vertices in $V(Q) {\setminus } X$ of degree at most one. For a node *t* of *T*, we denote by $\bar{H}_t^2$ the 2-center of $(H_t,X_t)$, where $H_t$ is the torso of $(T,\mathcal {X})$ at *t*. Let us denote $|\bar{H}_t^2|$ by ${\text {tor}}_2(t)$.

#### Definition 4

The slim width of a tree-cut decomposition $(T,\mathcal {X})$ of a graph *G* is ${\text {stcw}}(T, \mathcal {X})=\max _{t\in V(T)}\{ {\text {adh}}(t), {\text {tor}}_2(t) \}$. The slim tree-cut width of *G*, or ${\text {stcw}}(G)$ in short, is the minimum slim width of $(T,\mathcal {X})$ over all tree-cut decompositions $(T,\mathcal {X})$ of *G*.

Observe that the difference in definitions of ${\text {tcw}}(G)$ and ${\text {stcw}}(G)$ is whether we dissolve the vertices of degree at most two or at most one in the torso in each node. At this point, it would be reasonable to ask what happens if we dissolve only isolated vertices (i.e., vertices of degree 0) from the torso. Naturally extending the notions of 2- and 3-center for a connected graph *Q* and $X\subseteq V(Q)$, we define the *1-center* of (*Q*, *X*) as the graph obtained from *Q* by deleting isolated vertices in $V(Q) {\setminus } X$. For a node *t* of *T*, we denote by $\bar{H}_t^1$ the 1-center of $(H_t,X_t)$, where $H_t$ is the torso of $(T,\mathcal {X})$ at *t*. Let us denote $|\bar{H}_t^1|$ by ${\text {tor}}_1(t)$.

#### Definition 5

The 0-width of a tree-cut decomposition $(T,\mathcal {X})$ of *G* is defined as $\max _{t\in V(T)}\{ {\text {adh}}(t),$ ${\text {tor}}_1(t) \}$. The 0-tree-cut width of *G*, or ${\text {tcw}}_0(G)$ in short, is the minimum 0-width of $(T,\mathcal {X})$ over all tree-cut decompositions $(T,\mathcal {X})$ of *G*.

It follows from the definitions that for any tree-cut decomposition $(T,\mathcal {X})$ of *G*, for each node *t* of *T*, ${\text {tor}}(t)\le {\text {tor}}_2(t) \le {\text {tor}}_1(t)$. In particular, the width of $(T,\mathcal {X})$ is upper-bounded by its slim width, while the latter does not exceed the 0-width of $(T,\mathcal {X})$.

#### Corollary 1

For any graph *G*, ${\text {tcw}}(G)\le {\text {stcw}}(G) \le {\text {tcw}}_0(G).$

The gaps in these inequalites can be arbitrarily large—and, more strongly, ${\text {tcw}}_0$ is a more restrictive parameter than ${\text {stcw}}$, which is in turn more restrictive than ${\text {tcw}}$. Indeed, for the comparison of ${\text {tcw}}_0$ and ${\text {stcw}}$ consider the class of stars which have slim tree-cut width 1. Let $S_r$ denote the star with *r* leaves (i.e., the complete bipartite graph $K_{1,r}$).

#### Lemma 2

For every positive integer $r\ge 1$, ${\text {tcw}}_0(S_{r^2})\ge r$.

#### Proof

Let $(T,\mathcal {X})$ be a tree-cut decomposition of $S_{r^2}$ of 0-width *k* where the bags of leaves are non-empty. Let *t* be the node of *T* such that $X_t$ contains the vertex of degree $r^2$. Observe that *t* has at most ${\text {tor}}_1(t)-|X_t|\le k-|X_t|$ children. For every child $t'$ of *t*, $Y_{t'}$ contains at most ${\text {adh}}(t')\le k$ vertices of $S_{r^2}$. In total, $Y_t$ contains at most $|X_t| +k \cdot ( k-|X_t|)\le k^2$ vertices of $S_{r^2}$. Together with at most ${\text {adh}}(t)\le k$ vertices outside of $Y_t$, $S_{r^2}$ has at most $k\cdot (k+1)$ vertices and hence $k\ge r$. $\square$

**Fig. 3:**
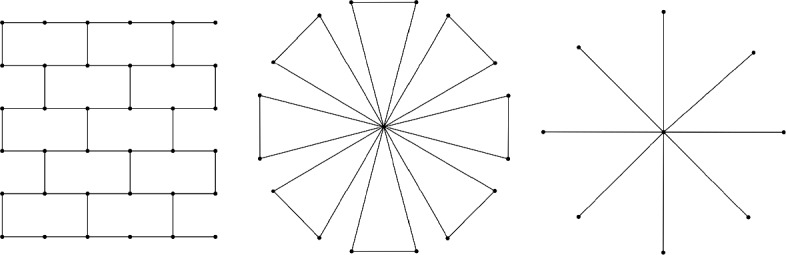
Illustrations of forbidden weak immersions for the graphs with bounded standard, slim or 0-tree-cut width. Left: 6-wall $H_6$, Middle: windmill $W_8$, Right: star $S_8$

To show the gap between ${\text {stcw}}$ and ${\text {tcw}}$, let us denote by $W_r$ the graph on $2r+1$ vertices consisting of *r* triangles sharing one vertex; here we call such graphs windmills, and refer to Fig. 3 later for an illustration. The class of windmills has tree-cut width 2 but, as the following lemma shows, unbounded slim tree-cut width.

#### Lemma 3

For every positive integer $r\ge 1$, ${\text {stcw}}(W_{r^2})\ge r$.

#### Proof

The case $r=1$ is straightforward. For $r\ge 2$, assume, to the contrary, that there exists a tree-cut decomposition $(T,\mathcal {X})$ of $W_{r^2}$ of slim width at most $r-1$. Let *t* be the node of *T* such that $X_t$ contains the vertex of degree $2r^2$. Without loss of generality, we assume that all the leaves of *T* have non-empty bags. Then the adhesion of any child $t'$ of *t* is at least two, as $Y_{t'}$ contains some vertex *v* of $W_{r^2}$ and the two edge-disjoint paths from *v* to the high-degree vertex in *t* each contribute to ${\text {adh}}(t')$. Hence, *t* has at most ${\text {tor}}_2(t)\le r-1$ children. Moreover, for every child $t'$ of *t*, $Y_{t'}$ intersects at most $\frac{r-1}{2}$ distinct triangles of $W_{r^2}$, since each such triangle contributes 2 to ${\text {adh}}(t')$. Hence, for every child $t'$ of *t*, $Y_{t'}$ contains at most $r-1$ vertices of $W_{r^2}$. In total, $Y_t\setminus X_t$ contains at most $(r-1)^2$ vertices of $W_{r^2}$. Since both ${\text {adh}}(t)$ and $|X_t|$ are upper-bounded by $r-1$ and the former bounds the number of vertices outside of $Y_t$ by $r-1$, this would mean that $W_{r^2}$ has at most $(r-1)^2+2r-2$ vertices, a contradiction with the definition of $W_{r^2}$. $\square$

Given a graph *G* and its nice tree-cut decomposition $(T,\mathcal {X})$ of width at most *k*, let us denote by $B_t^{(2)}$ the set of children of *t* from $B_t$ with adhesion precisely two; notice that $B_t^{(2)}$ does not necessarily contain all children of *t* with adhesion precisely two, since some may lie in $A_t$. Observe that for every fixed vertex *t* of *T*, if *x* is an element of 2-center of the torso at *t* and $x \not \in X_t$, then *x* corresponds either to the parent of *t* in *T* or to some child of *t* from $A_t\cup B_t^{(2)}$. Hence ${\text {tor}}_2(t) \le 1+|X_t|+|A_t|+|B_t^{(2)}|\le 3k+2 + |B_t^{(2)}|$.

#### Corollary 2

Let *G* be a graph with tree-cut decomposition $(T,\mathcal {X})$ of width at most *k*. Then for each node *t* of *T* it holds that $|B_t^{(2)}|\ge {\text {tor}}_2(t)-3k-2.$

### Weak Immersions

Naturally extending the result of Wollan for tree-cut width [38], we show that both slim and 0-tree-cut width are closed under weak immersions.

#### Theorem 1

If *G* and *H* are graphs such that $H\le _I G$ then ${\text {stcw}}(H)\le {\text {stcw}}(G)$ and ${\text {tcw}}_0(H)\le {\text {tcw}}_0(G)$.

#### Proof

It is sufficient to proof the statement when *H* is obtained from *G* by precisely one edge deletion, isolated vertex deletion or lifting a pair of edges. Let $(T,\mathcal {X})$ be a tree-cut decomposition of *G* of minimum slim (or 0-) width. Then $(T,\mathcal {X})$ is also a tree-cut decomposition of $G\setminus e$ for any edge *e* of *G* with the same or smaller slim (0-) width. Similarly for the isolated vertex deletion: we just need to delete the vertex from the corresponding bag. It remains to consider the case $H=G\setminus \{(x,y),(y,z)\} \cup (x,z)$ for some $(x,y),(y,z) \in E(G)$.

Notice that the lifting operation doesn’t increase adhesion of any node *t* of *T*: if the edge (*x*, *z*) has endpoints in different connected components of $T \setminus e(t)$ then so does at least one of the edges (*x*, *y*) or (*y*, *z*). To see that ${\text {tor}}_2(t)$ and ${\text {tor}}_1(t)$ do not increase either, denote by $Q_G$ and $Q_H$ the torsos at *t* in $(T, \mathcal {X})$ for graphs *G* and *H* correspondingly. Every vertex of $Q_G$ corresponds to a non-empty subset of the vertices of *G*. Depending on how the vertices *x*, *y* and *z* are split among these subsets, it holds that either $E(Q_H)\subseteq E(Q_G)$ (which yields the same or smaller 1-center and 2-center) or $Q_H$ is obtained from $Q_G$ by splitting a pair of edges. For the latter, observe that $v\in V(Q_G)\setminus X_t$ is not in the 2-center of $(Q_G, X_t)$ if and only if *v* belongs to some induced subtree of $Q_G$ connected to the rest of $Q_G$ by at most one edge. It is not hard to see that lifting the pair of edges preserves the property. For the 1-center the situation is even simplier: isolated vertices of $Q_G$ remain isolated. $\square$

Recall that the weak immersion relation $\le _I$ is a transitive, reflexive and antisymmetric relation on the set of finite graphs, i.e., a partial order. The previous theorem showed that ${\text {stcw}}$ is monotone with respect to $\le _I$. Our next goal is to find graphs of simple structure but large slim (or 0-) tree-cut width, such that forbidding them as weak immersions bounds the corresponding width of a graph. Wollan in [38] characterized such graphs for tree-cut width. Namely, he established the following dichotomy:

#### Theorem 2

(a) If *G* is a graph such that $H_{2r^2}\le _I G$ for some $r\ge 3$, then ${\text {tcw}}(G)\ge r$. (b) There exists a function $f:\mathbb {N}\rightarrow \mathbb {N}$ such that if ${\text {tcw}}(G)\ge f(r)$, then $H_r\le _I G$, $r\in \mathbb {N}$.

Here $H_r$ denotes the *r*-wall, the graph which can be obtained from the $r \times r$ grid by deleting every second vertical edge in each row, see [38] for the definition and Fig. 3 for an illustration. We are going to complete the family of excluded immersions to provide similar characterizations for 0-tree-cut width and slim tree-cut width. Recall that the families of stars $S_r$ and windmils $W_r$ have unbounded 0- and slim tree-cut width, respectively (Lemmas 2 and 3). Combining this with Theorem 1, we immediatedly obtain:

#### Lemma 4

For every positive integer *r*, if ${\text {stcw}}(G) < r$ $({\text {tcw}}_0(G) < r)$, then *G* does not admit $W_{r^2}$ $(S_{r^2}$, respectively) as a weak immersion.

As we will show in the remainder of this subsection, excluding $W_r$ ($S_r$) as a weak immersion along with $H_r$ is actually sufficient to bound slim tree-cut width (0-tree-cut width).

#### Theorem 3

If *G* is a graph such that $H_{2r^2}\le _I G$ for some $r\ge 3$ or $S_{r^2}\le _I G$ for some $r\ge 1$, then ${\text {tcw}}_0(G)\ge r$. Moreover, there exists a function $h:\mathbb {N}\rightarrow \mathbb {N}$ such that if ${\text {tcw}}_0(G)\ge h(r)$, then $H_r\le _I G$ or $S_{r}\le _I G$.

#### Proof

If $H_{2r^2}\le _I G$ for some $r\ge 3$, we have that ${\text {tcw}}(G)\ge r$ by Theorem 2 and hence ${\text {tcw}}_0(G)\ge r$. In case $S_{r^2}\le _I G$, the lower bound follows from Lemma 4.

Let *f* be the function given by Theorem 2. We define *h* by setting $h(r)=r\cdot f(r)+3\cdot f(r)+2$. Assume that *G* is a graph such that ${\text {tcw}}_0(G) \ge h(r)$. If ${\text {tcw}}(G) \ge f(r)$, we immediatedly conclude that $H_r\le _I G$ by Theorem 2. Otherwise, let $(T,\mathcal {X})$ be a nice tree-cut decomposition of *G* of width at most *f*(*r*) with leaves having non-empty bags. There exists a node *t* of *T* such that ${\text {tor}}_1(t)\ge h(r)$, in particular, $B_t\ge r \cdot f(r)$. As the size of $X_t$ is at most *f*(*r*), some vertex of $X_t$ has degree of at least *r* and hence $S_{r}\le _I G$. $\square$

Before providing similar characterization for slim tree-cut width, we introduce a simple technical modification of tree-cut decompositions, which will also be used later for establishing the connection between slim tree-cut width and edge-cut width. The aim is, roughly speaking, to avoid the situation where a thin child has adhesion 2, even though it consists of two completely independent components each of which could be a thin child of adhesion 1. Formally, let $(T, \mathcal {X})$ be a nice tree-cut decomposition of *G*. We say that a node *t* with parent $t'$ in *T* is *decomposable* if the following conditions hold:

- $t\in B_{t'}$ and there exist two edges $e_1$ and $e_2$ between $G_t$ and $G\setminus G_t$ in *G*;
- the endpoints of $e_1$ and $e_2$ in $G_t$ belong to different connected components of $G_t$.

#### Lemma 5

Any nice tree-cut decomposition of *G* can be transformed into a nice tree-cut decomposition of the same tree-cut width with no decomposable nodes.

#### Proof

Let $(T', \mathcal {X}')$ be a nice tree-cut decomposition of *G* with at least one decomposable node. Let *t* be a decomposable node of $T'$ with minimum distance to the root, and let $e_1$ and $e_2$ be the edges between $G_t$ and $G\setminus G_t$ in *G*. We create a copy $T'_{t^*}$ of the rooted subtree $T'_t$ where the copy of $s\in T'_t$ is $s^*\in T'_{t^*}$. We then connect $t^*$ to the parent of *t*. Let $G_1$ be the connected component of $G_t$ containing an endpoint of $e_1$. For every $s \in V(T'_t)$ we set $X_s=X'_s \cap V(G_1)$ and $X_{s^*}=X'_s{\setminus } X_s$. For the rest of nodes *s* of $T'$ we set $X_s=X'_s$. Finally, we exhaustively remove empty bags which are leaves and denote the obtained tree by *T*. Observe that the resulting decomposition $(T,\mathcal {X})$ is nice and its width is not greater than the width of $(T', \mathcal {X}')$. Moreover, our transformation doesn’t create any decomposable nodes outside of subtrees rooted in *t* and $t^*$; both *t* and $t^*$ have an adhesion of one and hence are not decomposable. Therefore, after a finite number of such steps we obtain some nice tree-cut decomposition of *G* of the same width but with no decomposable nodes. $\square$

Further, as a technical term, we will refer to nice decompositions with no decomposable nodes as *very nice* decompositions.

#### Corollary 3

Every tree-cut decomposition can be transformed into a very nice tree-cut decomposition in quartic time, without increasing the width.

#### Proof

Let $(T'',\mathcal {X}'')$ be a tree-cut decomposition of *G* of width *k*. We transform $(T'',\mathcal {X}'')$ into a nice tree-cut decomposition $(T',\mathcal {X}')$ of width at most *k* (this can be done in cubic time, see [16] for details). Further, we apply Lemma 5 on $(T',\mathcal {X}')$. This requires at most quartic time, since every node of $T'$ is decomposed at most once and every such decomposition can be performed in cubic time. Then the resulting decomposition $(T,\mathcal {X})$ is very nice and has width of at most *k*. $\square$

With this transformation in hand, we are now ready to fully characterize forbidden weak immersions for graphs of bounded slim tree-cut width.

#### Theorem 4

If *G* is a graph such that $H_{2r^2}\le _I G$ for some $r\ge 3$ or $W_{r^2}\le _I G$ for some $r\ge 1$, then ${\text {stcw}}(G)\ge r$. Moreover, there exists a function $g:\mathbb {N}\rightarrow \mathbb {N}$ such that if ${\text {stcw}}(G)\ge g(r)$, then $H_r\le _I G$ or $W_{r}\le _I G$.

#### Proof

If $H_{2r^2}\le _I G$ for some $r\ge 3$, we have that ${\text {tcw}}_2(G)\ge r$ by Theorem 2 and hence ${\text {stcw}}(G)\ge r$. In case $W_{r^2}\le _I G$, the lower bound follows from Lemma 4.

Let *f* be the function given by Theorem 2. We define *g* by setting $g(r)=2r\cdot f^2(r)+3\cdot f(r)+2$. Assume that *G* is a graph such that ${\text {stcw}}(G) \ge g(r)$. If ${\text {tcw}}(G) \ge f(r)$, we immediatedly conclude that $H_r\le _I G$ by Theorem 2. Otherwise, by Corollary 3 there exists a very nice tree-cut decomposition $(T,\mathcal {X})$ of *G* of width at most *f*(*r*). Let us pick a node *t* of *T* such that ${\text {tor}}_2(t)\ge g(r)$. By Corollary 2 we have that $|B_t^{(2)}| \ge g(r)-3 \cdot f(r)-2 = 2r\cdot f^2(r)$. Since $(T,\mathcal {X})$ is very nice, all the children of *t* in $B_t^{(2)}$ are non-decomposable. Recall that for every $t'\in B_t^{(2)}$, the neighbourhood of $Y_{t'}$ in *G* is a one- or two-element subset of $X_t$, and hence $Y_{t'}$ provides a path between some (possibly equal) vertices of $X_t$. As the size of $X_t$ is at most *f*(*r*), *G* contains either 2*r* cycles intersecting in one vertex of $X_t$ or 2*r* paths between two vertices of $X_t$. Since every such pair of paths can be transformed into a cycle by lifting the pair of their first edges, in both cases we have $W_{r}\le _I G$. $\square$

### *k*-Edge Sums

Another natural property Wollan [38] established for tree-cut width is that the parameter is closed under the operation of taking *k*-edge sum for small *k*. Specifically, he proved:

#### Lemma 6

([38]) Let *G*, $G_1$, and $G_2$ be graphs such that $G = G_1\oplus _k G_2$. If $G_j$ has a tree-cut decomposition $(T_j, \mathcal {X}_j)$ for $j = 1,2$, then *G* has a tree-cut decomposition $(T,\mathcal {X})$ such that ${\text {adh}}(T,\mathcal {X})=\max \{k,{\text {adh}}(T_1,\mathcal {X}_1), {\text {adh}}(T_2, \mathcal {X}_2)\}$. Moreover, for every $t\in V(T)$, the torso $H_t$ of *t* in $(T,\mathcal {X})$ is isomorphic to the torso of some vertex of $(T_1,\mathcal {X}_1)$ or $(T_2,\mathcal {X}_2)$.

Based on this result for optimal decompositions $(T_1,\mathcal {X}_1)$ and $(T_2,\mathcal {X}_2)$, we immediatedly obtain the upper bound on 0- and slim tree-cut width for *k*-edge sums:

#### Corollary 4

Let *G*, $G_1$ and $G_2$ be graphs such that $G = G_1\oplus _k G_2$. Then it holds that ${\text {stcw}}(G) \le \max \{k, {\text {stcw}}(G_1), {\text {stcw}}(G_2)\}$ and moreover ${\text {tcw}}_0(G) \le \max \{k,{\text {tcw}}_0(G_1), {\text {tcw}}_0(G_2)\}$.

In particular, if both $G_1$ and $G_2$ have 0-, slim or standard width of at most $\omega$ and $k \le \omega$, we may conclude that the corresponding width of *G* is at most $\omega$.

## Alternative Characterizations

In this section, we study alternative characterizations of slim tree-cut width and 0-tree-cut width. In particular, we observe that the latter is asymptotically equivalent to maximum degree plus treewidth. This provides an interesting connection between tree decompositions and tree-cut decompositions, but essentially rules out its study as a means of establishing novel tractability results. For slim tree-cut width, however, we obtain a characterization that ties it to the previously studied edge-cut width and has algorithmic implications.

### Characterization of 0-Tree-Cut Width

Wollan [38] showed that a bound on the treewidth and maximum degree implies a bound on the tree-cut width of a graph:

#### Proposition 2

Let *G* be a graph with maximal degree *d* and treewidth *w*. Then there exists a tree-cut decomposition of adhesion at most $(2w+2)d$ such that every torso has at most $(d+1)(w+1)$ vertices.

In particular, as ${\text {tor}}_1(t)\le |H_t|\le (d+1)(w+1) \le (2w+2)d$ for every node *t* of *T*, we have ${\text {tcw}}_0(G)\le (2w + 2)d$. In the following proposition, we show that the converse is true as well: bounded ${\text {tcw}}_0$ implies bounded treewidth and maximum degree of a graph.

#### Proposition 3

Let *G* be a graph with ${\text {tcw}}_0(G) = k$. Then every vertex of *G* has degree of at most $k^2+2k$ and ${\text {tw}}(G)\le 2k^2+3k$.

#### Proof

By Lemma 1 and Corollary 1 we have that ${\text {tw}}(G)\le 2 {\text {tcw}}(G)^2+3 {\text {tcw}}(G) \le 2k^2+3k$. Since ${\text {tcw}}_0(G) = k$, Lemma 2 implies that *G* does not contain $S_{(k+1)^2}$ as a weak immersion, in particular, degree of any vertex of *G* is at most $k^2+2k$. $\square$

#### Corollary 5

0-tree-cut width is asymptotically equivalent to maximum degree plus treewidth.

This also implies an immediate connection to cutwidth – another edge-cut based parameter, defined in terms of linear orderings. A *linear ordering* of *G* is a bijective mapping $f: V \rightarrow \{1, \dots , n\}$. The *cut* at vertex *v* with respect to *f* (denoted ${\text {cut}}(v)$) is $|\{(u,w)\in E~|~f(u)\le f(v) < f(w)\}|$. The cutwidth of a linear ordering *f* is $\max _{v\in V(G)}{\text {cut}}(v)$. The *cutwidth* of *G* is the minimum cutwidth of *f* over all linear orderings *f* of *G*.

Observe that the degree of every vertex *v* of *G* is at most two times the cutwidth of *G*: neighbours of *v* that go after *v* in the ordering contribute to ${\text {cut}}(v)$, while the rest of the neighbours contribute to ${\text {cut}}(w)$ where $f(w)=f(v)-1$. It is known that both the pathwidth and the treewidth of a graph is upper-bounded by its cutwidth [29], and in particular this implies that the class of binary trees has unbounded cutwidth. Hence, cutwidth is a strictly more restrictive parameter than ${\text {degtw}}$ (and hence ${\text {tcw}}_0$).

### Characterization of Slim Tree-Cut Width

Recall that edge-cut width is a parameter that is defined over spanning trees in the input graph *G*, which serve as the corresponding decompositions. Let us now consider a slight generalization of this where we consider not only spanning trees over *G*, but of any supergraph of *G*. Such a generalization would—unlike edge-cut width itself—trivially be closed under both vertex and edge deletion. For our considerations, let us denote this parameter *super edge-cut width* (${\text {sec}}(G)$):

$$\begin{aligned}{\text {sec}}(G)=\min \{{\text {ecw}}(H, T)~|~H\supseteq G \text { and } T\text { is a spanning forest of }H\}.\end{aligned}$$

If $H\supseteq G$ is a supergraph of *G* and *T* is a spanning forest of *H* such that ${\text {ecw}}(H, T) \le k$, we say that *T* *witnesses* ${\text {sec}}(G) \le k$. Observe that there always exists a connected witness, i.e., a tree. Indeed, if *H* consists of $m>1$ connected components, we can arbitrarily extend it to a connected graph $H^*$ by adding $m-1$ edges. The addition of these edges to *T* then results in the tree $T^*$ witnessing ${\text {sec}}(G)\le k$. Moreover, notice that any witness of ${\text {ecw}}(G)\le k$ is also a witness of ${\text {sec}}(G)\le k$.

#### Corollary 6

For every graph *G*, ${\text {sec}}(G)\le {\text {ecw}}(G)$.

However, graphs of constant super edge-cut width can have arbitrarily large edge-cut width, as will become clear at the end of the section. A slight modification of the proof of Proposition 1 yields:

#### Proposition 4

For every graph *G*, ${\text {tcw}}(G)\le {\text {sec}}(G)$.

#### Proof

Let *Q* be the supergraph of *G* and let *T* be the spanning tree of *Q* such that ${\text {ecw}}(Q,T)={\text {sec}}(G)$. We construct a tree-cut decomposition $(T, \mathcal {X})$ of *G* where each bag contains at most one vertex, notably by setting $X_t=\{t\}$ for each $t\in V(G)$ and $X_t=\emptyset$ for each $t\in V(Q)\setminus V(G)$. Fix any node *t* in *T* other than the root, let *u* be the parent of *t* in *T*. All the edges of $G\setminus ut$ with one endpoint in the rooted subtree $T_t$ and another outside of $T_t$ belong to $E^{Q,T}_{loc}(t)$, so ${\text {adh}}_T(t)\le |E^{Q,T}_{loc}(t)|+1 \le {\text {sec}}(G)$.

Let $H_t$ be the torso of $(T, \mathcal {X})$ in *t*, then $V(H_t)=X_t \cup \{z_1...z_l\}$ where $z_i$ correspond to connected components of $T {\setminus } t$, $i\in [l]$. In ${\tilde{H}}_t$, only $z_i$ with degree at least 3 are preserved. But all such $z_i$ are the endpoints of at least two edges in $|E^{Q,T}_{loc}(t)|$, so ${\text {tor}}(t)=|V({\tilde{H}}_t)|\le 1+ |E^{Q,T}_{loc}(t)| \le {\text {sec}}(G)$. Thus ${\text {tcw}}(G)\le {\text {sec}}(G)$. $\square$

To represent a deeper connection between tree-cut decompositions and super edge-cut width, it will be convenient to work with very nice decompositions introduced in Sect. 3.2.

#### Proposition 5

Let $(T, \mathcal {X})$ be a very nice tree-cut decomposition of *G* of width at most *k*. Then for each node *t* of *T*, $|B_t^{(2)}| \le k \cdot {\text {sec}}(G)$. In particular, ${\text {stcw}}(G) \le {\text {sec}}(G)^2+4 \cdot {\text {sec}}(G)$.

#### Proof

**Fig. 4:**
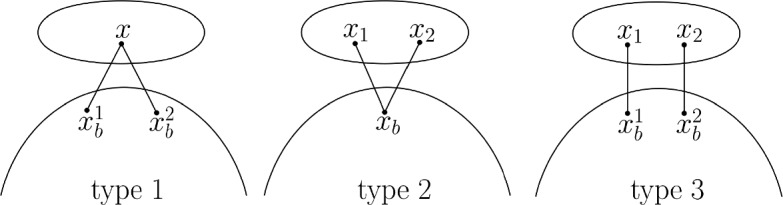
Possible configurations of edges between thin child $b\in B_t^{(2)}$ and its parent *t*

Assume that $T^*$ is a spanning tree of $H\supseteq G$ such that ${\text {sec}}(G)={\text {ecw}}(H, T^*)$. For any node *t* of *T* and $b\in B_t^{(2)}$, *b* has one of three types (see Fig. 4):

1. $N(Y_b)=\{x\}$ for some $x \in X_t$, *x* is connected to distinct $x_b^1$ and $x_b^2$ from $Y_b$;
2. $N(Y_b)=\{x_1,x_2\}$ for $x_1\ne x_2$, $x_1$ and $x_2$ are connected to the same $x_b\in Y_b$;
3. $N(Y_b)=\{x_1,x_2\}$ for $x_1\ne x_2$, $x_1$ and $x_2$ are connected to distinct $x_b^1$ and $x_b^2$ from $Y_b$ correspondingly;

Let us start with the first type. If $x_b^ix$ doesn’t belong to $T^*$ for $i=1$ or $i=2$, then $x_b^ix \in E^{H,T^*}_{loc}(x)$. Otherwise, $x_b^1$ and $x_b^2$ are connected via *x* in $T^*$. Then $T^*[Y_b]$ has precisely two connected components. As *b* is not decomposable, there exists a path *p* between $x_b^1$ and $x_b^2$ in $G_b$ containing precisely one edge outside of $T^*$. This edge contributes to $E^{H,T^*}_{loc}(x)$.

As $T^*$ is a tree, there can be at most $|X_t|-1\le k-1$ thin children *b* of the second type such that $x_b$ is adjacent to two elements of $X_t$ in $T^*$. For the rest of *b* of the second type, there exists $x\in X_t$ such that $xx_b\in G{\setminus } T^* \subseteq H{\setminus } T^*$ and therefore $xx_b \in E^{H,T^*}_{loc}(x)$.

Let *b* be a thin node of the third type. If $x^b_1$ and $x^b_2$ are connected via a path in $T^*[Y_b]$, we can apply the same argument as for the second type. Otherwise, $T^*[Y_b]$ has precisely two connected components and, analogously to the first type, there exists an edge in $G_b\cup \{x_1 x^b_1, x_2 x^b_2\}$ that belongs to $E^{H,T^*}_{loc}(x_1)$.

To conclude, any node of $B_t^{(2)}$ either increases $E^{H,T^*}_{loc}(x)$ for some $x\in X_t$ or creates a path in $T^*$ between two vertices of $X_t$. Since $T^*$ is a tree, $|X_t|\le k$ and $|E^{H,T^*}_{loc}(x)|\le {\text {sec}}(G)-1$ for every $x\in X_t$, the size of $B_t^{(2)}$ is at most $(k-1)+\sum _{x\in X_t}|E^{H,T^*}_{loc}(x)|\le k\cdot {\text {sec}}(G)-1$. Then ${\text {tor}}_2(t) \le |A_t|+|X_t|+1+|B_t^{(2)}| \le 3k+1+k\cdot {\text {sec}}(G) \le k\cdot ({\text {sec}}(G)+4)$. Since the bound holds for every node *t* of *T*, we may conclude that the slim width of $(T, \mathcal {X})$ is at most $k\cdot ({\text {sec}}(G)+4)$. By Proposition 4 and Corollary 3, there exists a very nice tree-cut decomposition of *G* of width $k\le {\text {sec}}(G)$, therefore ${\text {stcw}}(G) \le {\text {sec}}(G)^2+4\cdot {\text {sec}}(G)$. $\square$

Hence, slim tree-cut with of any graph is upper-bounded by a quadratic function of its super edge-cut width. Next, we show that the converse statement holds as well:

#### Proposition 6

For every graph *G*, ${\text {sec}}(G)\le 3\cdot ({\text {stcw}}(G)+1)^2$. Moreover, given a tree-cut decomposition of *G* of slim width *k*, it is possible to compute a supergraph $Q\supseteq G$ and its spanning tree *T* witnessing ${\text {sec}}(G)\le 3(k+1)^2$ in cubic time.

#### Proof

Let $(T_0,\mathcal {X}_0)$ be a tree-cut decomposition of *G* of slim width *k*. We start by transforming it into a nice tree-cut decomposition $(T,\mathcal {X})$ in cubic time as in [16]. The transformation procedure acts on the 2-centers of torsos only by contracting some edges. Recall that $v\in V(H_t)\setminus X_t$ is not in the 2-center of $(H_t, X_t)$ if and only if *v* belongs to some induced subtree of $H_t$ connected to the rest of $H_t$ by at most one edge. Since contracting an edge either preserves the property or merges *v* with some other vertex, it doesn’t increase ${\text {tor}}_2(t)$ for any node *t* of *T*. In particular, the slim width of $(T,\mathcal {X})$ is at most *k*.

Let $\Omega \subseteq \mathcal {X}$ be the set of empty bags of $(T,\mathcal {X})$, we construct $Q\supseteq G$ along with its tree-cut decomposition $(T,\mathcal {X'})$ as follows. Firstly, we add to *G* vertices $v_t$ for every $t\in \Omega$. We define $X'_t=\{v_t\}$ if $X_t=\emptyset$ and $X'_t=X_t$ otherwise. For every node $t\in T$, construct an arbitrary tree $T^*_t$ over $X'_t$ and add its edges to *Q*. Further, we process every edge $e=pt \in E(T)$ such that *p* is the parent of *t* in *T* and either $N(Y_t)\not \subseteq X_t$ or ${\text {adh}}(t)>1$ as follows. If *G* doesn’t contain an edge between $X_t'$ and $X'_p$, we add to *E*(*Q*) arbitrary edge with endpoints in $X_t'$ and $X'_p$. This increases the adhesion of *e* by at most one.

Now we proceed to the choice of the spanning tree $T^*$ in *Q*. For every $t\in T$ other then the root, let *p* be the parent of *t* in *T*. If ${\text {adh}}(t)=1$ and $N(Y_t) \subseteq X_t$, we denote by $e_t$ the unique edge between $Y'_t$ and $X'_p$ in *Q*. Otherwise, let $e_t$ be arbitrary edge of *Q* with endpoints in $X_t'$ and $X'_p$. We then construct $T^*$ by gluing together all $T^*_t$ via edges $e_t$: $T^*=(\cup _{t\in V(T)}T^*_t)\bigcup (\cup _{t\in V(T){\setminus } r} \{e_t\})$. Obviously the construction can be performed in cubic time; we will show that ${\text {sec}}(Q, T^*) \le 3(k+1)^2$.

To this end, fix any node *t* of *T* and $$x\in X'_t$$ and denote $$E_{loc}(x)=E_{loc}^{Q,T^*}(x)$$. If $$T^*$$ contains more than one edge between $$Y'_t$$ and rest of $$T^*$$, then all but one of them are the unique edges connecting $$Q'_q$$ to the rest of *Q* for some descendants *q* of *t* in *T*. Hence, they don’t belong to any path in $$T^*$$ between the endpoints of some feedback edge $$e\in E(Q){\setminus } E(T^*)$$. Therefore, every edge of $$ E_{loc}(x)$$ has at least one endpoint in $$Y'_t$$. The number of edges in $$E_{loc}(x)$$ with both endpoints in $$X_t'$$ is at most $$|X_t'|\cdot (|X_t'|-1)\le k\cdot (k-1)$$. Every edge with one endpoint in $$X_t'$$ and another outside of $$Y_t'$$ contributes to the adhesion of *t* in $$(T,\mathcal {X'})$$, so their number is bounded by $$k+1$$.

Finally, if $e=yz \in E_{loc}(x)$ contains an endpoint *y* in $Y'_t {\setminus } X'_t$, then $y\in Y'_q$ for some child *q* of *t*. Then *Q* contains a cycle intersecting $Y'_q$ and $x\in X_t$. In particular, by construction of *Q* we may conclude $q\in A_t \cup B_t^{(2)}$ w.r.t. the decomposition $(T,\mathcal {X})$. By the same arguments as for the node *t*, we conclude that at most one edge between $Y'_q$ and the rest of $T^*$ belongs to any path in $T^*$ between the endpoints of some feedback edge $e\in E(Q){\setminus } E(T^*)$, so $z\not \in Y'_q$ and *e* contributes to the adhesion of *q* in $(T,\mathcal {X'})$. In particular, $E_{loc}(x)$ contains at most ${\text {adh}}(q)+1$ edges with an endpoint in $Y'_q$. In total, at most $\max _{q\in A_t}({\text {adh}}(q)+1)\cdot |A_t| +\max _{q\in B_t^{(2)}}({\text {adh}}(q)+1)\cdot |B_t^{(2)}| \le (k+1)(2k+1)+3k=2k^2+6k+1$ edges in $E_{loc}(x)$ have an endpoint in $Y'_{t}\setminus X'_t$, so $|E_{loc}(x)|\le k\cdot (k-1)+(k+1)+2k^2+6k+1 =3k^2+6k+2$ and hence $\sec (Q, T^*)\le 3k^2+6k+3 =3(k+1)^2$. $\square$

#### Corollary 7

${\text {sec}}$ and ${\text {stcw}}$ are asymptotically equivalent.

**Fig. 5:**
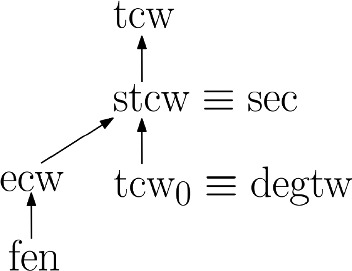
Position of slim and 0-tree-cut width in the hierarchy of edge-cut based parameters. An arrow from *p* to *q* represents the fact that *p* is more restrictive than *q*, while asymptotic equivalence is depicted by $\equiv$

The results of this section are summarized in Fig. 5. In particular, the graph family provided in [4, Lemma 2] shows that graphs of constant super edge-cut width may have arbitrarily large edge-cut width.

## Approximating Slim Tree-Cut Width

In this section we show how to efficiently construct a tree-cut decomposition of a graph *G* with slim width bounded by a cubic function of its optimal value ${\text {stcw}}(G)$. As a starting point for our approximation, we use the following result of Kim, Oum, Paul, Sau and Thilikos:

### Theorem 5

([28]) There exists an algorithm that, given a graph *G* and $\omega \in \mathbb {N}$, either outputs a tree-cut decomposition of *G* with width at most $2\omega$ or correctly reports that no tree-cut decomposition of *G* with width at most $\omega$ exists in $2^{\mathcal {O}(\omega ^2 \cdot log \omega )}\cdot n^2$ steps.

As an observant reader might have already noticed, if *G* has bounded slim tree-cut width, it imposes some restrictions on the structure of possible decompositions of *G* of small (standard) tree-cut width. This fact enables us to construct an efficient approximation for ${\text {stcw}}(G)$.

### Theorem 6

There exists an algorithm that, given a graph *G* and $\omega \in \mathbb {N}$, either outputs a tree-cut decomposition of *G* with slim width at most $6(\omega +1)^3$ or correctly reports that no tree-cut decomposition of *G* with slim width at most $\omega$ exists in $2^{\mathcal {O}(\omega ^2 \cdot log \omega )}\cdot n^4$ steps.

### Proof

Given a graph *G* and $\omega \in \mathbb {N}$, let us run the algorithm from Theorem 5. If it reports that ${\text {tcw}}(G)>\omega$, we may conclude that ${\text {stcw}}(G)>\omega$ by Corollary 1. In case the algorithm returns a tree-cut decomposition $(T', \mathcal {X}')$ of width at most $2 \omega$, we invoke Corollary 3 to transform this decomposition into a very nice decomposition $(T, \mathcal {X})$ of the same width in at most quartic time. By Proposition 5, we have that $|B_t^{(2)}|\le 2\omega \cdot {\text {sec}}(G)$ for each node *t* of *T*. If for some node *t* the size of $B_t^{(2)}$ exceeds $6\omega \cdot (\omega +1)^2$, then ${\text {sec}}(G)>3(\omega +1)^2$ and by Proposition 6 we may correctly report that ${\text {stcw}}(G)>\omega$. Otherwise, ${\text {tor}}_2(t)\le 1+|X_t|+|A_t|+|B_t^{(2)}|\le 1+2\omega +(4 \omega + 1)+6\omega \cdot (\omega +1)^2 \le 6(\omega +1)^3$ for any node *t* of *T*. Hence, the slim width of $(T, \mathcal {X})$ is at most $6(\omega +1)^3$. $\square$

## Discussion of Algorithmic Applications

Having established its structural properties, we now turn to the algorithmic aspects of slim tree-cut width. Here, Corollary 7 shows that instead of using a tree-cut decomposition of the input graph *G* to design fixed-parameter algorithms—as was done in past dynamic programming algorithms that utilized tree-cut width—we can perform dynamic programming along a spanning tree *T* of a supergraph *Q* of *G*. Both *Q* and *T* can be computed from *G* in a pre-processing stage by using Proposition 6, and using a spanning tree instead of a tree-cut decomposition typically leads to significantly more concise (and conceptually cleaner) algorithms.

The cost for this simplification is the quadratic gap between the widths of these decompositions. We note that this situation is somewhat analogous to how one still typically uses clique-width [7] as a general and easy-to-use parameterization for various problems (especially when aiming for instances with higher edge-densities), even though rank-width [35] and Boolean-width [6] are asymptotically equivalent parameterizations which have been shown to yield more efficient algorithms [15]—there, the gap is even exponential.

Recall that a number of problems which remain ${{{\textsf {W}}}} [1]$-hard w.r.t. tree-cut width have recently been shown to be fixed-parameter tractable when parameterized by edge-cut width [4, 18], via explicit dynamic programming algorithms which proceed along the spanning tree of the input graph. While the functional gap between edge-cut width and super edge-cut width (and, analogously, slim tree-cut width) may be arbitrarily large, it is not difficult to see that each of the algorithms provided in those papers can be straightforwardly lifted to fixed-parameter algorithms w.r.t. super edge-cut width. Indeed, the only amendment one needs to make is to deal with the presence of “ghost” edges and vertices which occur in the spanning tree but not in the graph, and the computation of the records in these algorithms can easily deal with such vertices and edges.

To provide a concrete illustration of how this can be done, let us revisit the dynamic programming algorithm for the Edge Disjoint Paths problem parameterized by edge-cut width [4, Theorem 2]. No change is needed to the records. When the algorithm attempts to compute the set of “valid records” for a vertex *v* from the sets of valid records for some of its children $v_1,\dots ,v_\psi$ in the spanning tree, the algorithm performs a branching step in which it considers all possible ways the paths can be routed between the subtrees rooted at these children (See the “If v is an internal node” paragraph in the proof). At this branching step, we simply discard all routings which use edges that are not present in *G*. The situation is no more complicated for the other considered problems—in essentially all cases, the change simply boils down to ignoring the vertices and edges which do not exist in *G*.

Hence, we obtain:

### Corollary 8

(Theorems 2–6 in [4], Theorems 6 and 14 in [18]) List Coloring, Precoloring Extension, Boolean Constraint Satisfaction, Edge Disjoint Paths, Bayesian Network Structure Learning, Polytree Learning, Minimum Changeover Cost Arborescence, and Maximum Stable Roommates with Ties and Incomplete Lists are fixed-parameter tractable w.r.t. slim tree-cut width.

In parallel to the above, the structural properties of slim tree-cut width also allow us to obtain tractability results for some of the mentioned problems directly, based only on the existence of fixed-parameter algorithms w.r.t. treewidth plus maximum degree (which we will hereinafter simply refer to as *degree treewidth*). Intuitively, we do so by showing that every graph of bounded slim tree-cut width can be decomposed into a tree of 2-connected blocks of bounded treewidth and degree, and this structure can be processed in a leaf-to-root fashion. We formalize below.

For a connected graph $G=(V,E)$, we say that $e\in E$ is a *bridge* if $G\setminus e$ is disconnected. We intruduce an equivalence relation $\equiv$ on *V* such that for any $v,w \in V$, $v\equiv w$ if and only if the shortest path between *v* and *w* in *G* doesn’t contain bridges. For $v\in V(G)$, let us denote by ${\bar{v}}$ the equivalence class containing *v*. We construct the *block decomposition* $\Upsilon _G$ of *G* as follows:

- the set of nodes $V(\Upsilon _G)$ is the set of equivalence classes $\{{\bar{v}} ~|~v\in V\}$,
- for every bridge $e=(v,w)$ of *G*, add an edge ${\bar{e}}=({\bar{v}}, {\bar{w}})$ to $E(\Upsilon _G)$.

While a block decomposition is well-defined for any graph and always forms a tree, in general it doesn’t provide any algorithmic insights. For instance, if there are no bridges in *G*, then $\Upsilon _G$ consists of a unique node containing all the vertices of *G*. But in terms of slim tree-cut width, we can establish the following property:

### Lemma 7

Let *G* be a graph such that ${\text {stcw}}(G)\le k$. Then, for every ${\bar{v}} \in V(\Upsilon _G)$, ${\text {tcw}}_0(G[{\bar{v}}])\le k$.

### Proof

Consider a tree-cut decomposition $(T, \chi )$ of $G[{\bar{v}}]$ of slim width at most *k*. By definition, the adhesion in each node *t* of *T* is at most *k*. $G[{\bar{v}}]$ is connected and doesn’t contain bridges, so for every node *t* both ${\text {tor}}_1(t)$ and ${\text {tor}}_2(t)$ are equal to the size of the torso at *t*, in particular ${\text {tor}}_1(t)={\text {tor}}_2(t) \le {\text {stcw}}(G[{\bar{v}}])\le k$ and hence ${\text {tcw}}_0(G[{\bar{v}}])\le k$. $\square$

Along with Proposition 3 this immediatedly provides upper bounds for the maximum degree and treewidth of any induced subgraph $G[{\bar{v}}]$:

### Corollary 9

For a graph *G* and every ${\bar{v}} \in V(\Upsilon _G)$, maximal degree of $G[{\bar{v}}]$ is at most ${\text {stcw}}(G)^2+2{\text {stcw}}(G)$ and ${\text {tw}}(G[{\bar{v}}])\le 2{\text {stcw}}(G)^2+3{\text {stcw}}(G)$.

Next, we describe how this result can be used to lift fixed-parameter tractability from degree treewidth to slim tree-cut width. Let us start from the Edge Disjoint Paths problem, which was shown to be FPT w.r.t. degree treewidth in [21].
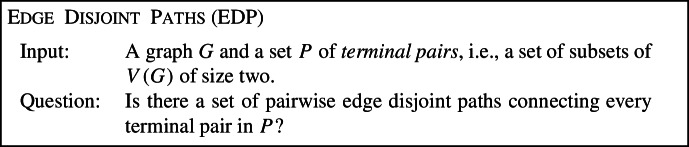


Consider an arbitrary instance (*G*, *P*) of $\textsc {EDP}$. Assume that $e\in E(G)$ is a bridge, and $G_1$ and $G_2$ are connected components of $G\setminus e$. If *P* contains at least two terminal pairs with one vertex in $G_1$ and another in $G_2$, we have a NO-instance, since any path connecting such a pair would be routed through *e*. Otherwise, if both endpoints of any terminal pair belong to the same connected component, we may solve the problem independently on $G_1$ and $G_2$, restricting the set of terminal pairs to their vertex sets. Finally, if there exists precisely one terminal pair $(v_1,v_2)\in P$ such that $v_1\in V(G_1)$ and $v_2\in V(G_2)$, then a path between $v_1$ and $v_2$ in a potential solution must contain the edge *e*. Let $x_i$ be the endpoint of *e* that belongs to $G_i$, $i=1,2$. To solve the original instance (*G*, *P*), it is sufficient to independently solve the instances $(G_i,P_i)$, where $P_i$ contains all the pairs from *P* with both endpoints in $V(G_i)$ plus the pair $(v_i,x_i)$, $i=1,2$.

Iteratively applying this argument for every bridge of *G*, we obtain the set of instances $(G[{\bar{v}}], P_v)$, ${\bar{v}} \in V(\Upsilon _G)$, such that (*G*, *P*) is a YES-instance if and only if every $(G[{\bar{v}}], P_v)$ is a YES-instance. Since by Corollary 9 the degree treewidth of each $G[{\bar{v}}]$ is bounded by a quadratic function of ${\text {stcw}}(G)$, we may now apply the fixed-parameter algorithm from [21].

### Corollary 10

Edge Disjoint Paths is fixed-parameter tractable w.r.t slim tree-cut width.

Some problems cannot be decomposed along bridges in such a simple way. In this case, instead of solving independent instances for each $G[{\bar{v}}]$, we try to iteratively compress the original instance, every time decreasing the number of nodes in the block decomposition. For example, let us consider the List Coloring problem. A *coloring* ${\text {col}}$ is a mapping from the vertex set of a graph to a set of colors; a coloring is *proper* if for every pair of adjacent vertices *a*, *b*, it holds that ${\text {col}}(a) \ne {\text {col}}(b)$. The problem can then be formulated as follows: 
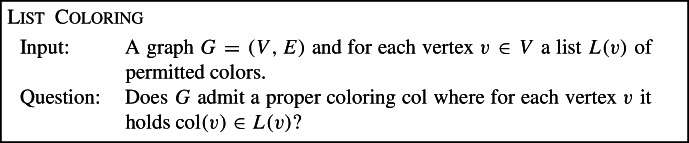


Consider an instance $\mathcal {I}=(G,L)$ of List Coloring. If $\Upsilon _G$ consists of a unique node, we conclude that *G* has bounded degree treewidth and therefore $\mathcal {I}$ can be solved in FPT time [16]. Otherwise, we show how to iteratively compress the leaves of $\Upsilon _G$.

Let ${\bar{v}} \in V(\Upsilon _G)$ be a leaf adjacent to ${\bar{w}}$, where $vw\in E(G)$. First, for each color $c \in L(v)$, we create an instance $\mathcal {I}_c= (G[{\bar{v}}],L_c)$ where $L_c$ coinsides with *L* on ${\bar{v}}$ except $L_c(v)=\{c\}$. Since $G[{\bar{v}}]$ has bounded degree treewidth, every such instance can be solved in FPT time [16]. Observe that $L'(v)=\{c \in L(v) ~|~\mathcal {I}_c$ is a YES-instance$\}$ is the set of all colors that *v* receives in some proper coloring of $G[{\bar{v}}]$ that agrees with *L*. If $L'(v)=\emptyset$, $\mathcal {I}$ is obviously a NO-instance. If the cardinality of $L'(v)$ is at least 2, removing ${\bar{v}}$ from *V*(*G*) results in an instance that is equivalent to $\mathcal {I}$: any coloring of $G\setminus {\bar{v}}$ can be extended to ${\bar{v}}$ so that *v* and *w* receive different colors. Finally, if $L'(v)$ contains a unique color *c*, we remove ${\bar{v}}$ from *V*(*G*) and, in case $c\in L(w)$, remove *c* from *L*(*w*). In each case, we reduce the number of nodes of the block decomposition by one. For the last node, we simply apply the ${{{\textsf {FPT}}}}$ algorithm from [16].

Precoloring Extension is another coloring problem from Table 1, where a graph is given with a partial coloring, and the task is to extend it to the proper coloring. As was mentioned in [4], this problem can be considered as a special case of List Coloring.

### Corollary 11

List Coloring and Precoloring Extension are fixed-parameter tractable w.r.t. slim tree-cut width.

To see one more example of lifting tractability from degree treewidth to slim tree-cut width, let us consider the classical constraint satisfaction problem over a Boolean domain [37]. An instance $\mathcal {I}$ of Boolean CSP is a tuple (*X*, *C*), where *X* is a finite set of *variables* and $\mathcal {C}$ is a finite set of *constraints*. Each constraint in $\mathcal {C}$ is a pair (*S*, *R*), where the *constraint scope* *S* is a non-empty sequence of distinct variables of *X*, and the *constraint relation* *R* is a relation over $\{0,1\}$ (given as a set of tuples) whose arity matches the length of *S*. An *assignment* is a mapping from the set *X* of variables to $\{0,1\}$. An assignment $\sigma$ satisfies a constraint $C = ((x_1, \ldots , x_n),R)$ if $(\sigma (x_1), \ldots , \sigma (x_n)) \in R$, and $\sigma$ satisfies the Boolean CSP instance if it satisfies all its constraints. An instance $\mathcal {I}$ is satisfiable if it is satisfied by some assignment. 
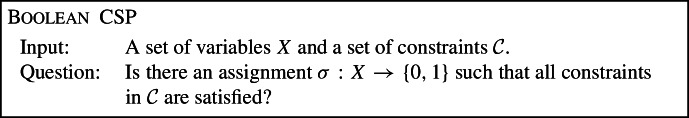


We represent this problem via the *incidence graph* *G*, whose vertex set is $X\cup \mathcal {C}$ and which contains an edge between a variable and a constraint if and only if the variable appears in the scope of the constraint. Boolean CSP is known to be fixed-parameter tractable when parameterized by the degree treewidth of *G* [37]. Similarly as before, to lift the result to slim tree-cut width, it is sufficient to provide a reduction compressing a leaf of $\Upsilon _G$ in a given instance $\mathcal {I}$.

Assume that ${\bar{v}} \in V(\Upsilon _G)$ is a leaf adjacent to ${\bar{c}}$, where *v*, *c* are the unique variable in ${\bar{v}}$ and constraint in ${\bar{c}}$, respectively, such that *v* appears in the scope of *c*. We create two instances $\mathcal {I}_0$ and $\mathcal {I}_1$, where $\mathcal {I}_i$ is obtained from $\mathcal {I}$ by restricting *G* to ${\bar{v}}$ and adding a new constraint with the scope (*v*) which is satisfied if and only if $\sigma (v)=i$. If both $\mathcal {I}_0$ and $\mathcal {I}_1$ are NO-instances, clearly $\mathcal {I}$ is a NO-instance as well. Otherwise, if both $\mathcal {I}_0$ and $\mathcal {I}_1$ are YES-instances, we simply remove from $\mathcal {I}$ all the variables and constraints that belong to ${\bar{v}}$ and remove *v* from the scope of *c* (i.e., we forget corresponding coordinate in the constraint relation). For correctness, observe that any satisfying assignment $\sigma$ of the modified instance can be extended to *v* to satisfy *c*. Then, if $\sigma (v)=i$, any solution to $\mathcal {I}_i$ provides an extension of $\sigma$ to the variables in ${\bar{v}}$. Finally, if $\mathcal {I}_i$ is a YES-instance and $\mathcal {I}_{1-i}$ is a NO-instance, we remove from $\mathcal {I}$ all the variables and constraints that belong to ${\bar{v}}$, same as before. But now, before removing *v* from the scope of *c*, we delete from the constraint relation of *c* all the tuples which assign to *v* the value $1-i$. This also results in an equivalent instance, since any sattisfying assignment for $\mathcal {I}$ must map *v* to *i*.

Next, let us consider the case when ${\bar{c}} \in V(\Upsilon _G)$ is a leaf adjacent to ${\bar{v}}$, and *c*, *v* are the unique constraint in ${\bar{c}}$ and variable in ${\bar{v}}$, respectively, such that *v* appears in the scope of *c*. For each $i\in \{0,1\}$, we try to satisfy all the constraints in ${\bar{c}}$ while assigning to *v* the value *i*. For this, we solve the instances $\mathcal {I}_0$ and $\mathcal {I}_1$ where $\mathcal {I}_i$ is obtained from $\mathcal {I}$ by deleting from *c* all the tuples that set *v* equal to $1-i$, removing *v* from the scope of *c* and then removing all the variables and constraints outside of ${\bar{c}}$. If both $\mathcal {I}_0$ and $\mathcal {I}_1$ are NO-instances, we conclude that $\mathcal {I}$ is a NO-instance. Otherwise, if both $\mathcal {I}_0$ and $\mathcal {I}_1$ are YES-instances, we simply remove from $\mathcal {I}$ all the variables and constraints that belong to ${\bar{c}}$. Finally, if $\mathcal {I}_i$ is a YES-instance and $\mathcal {I}_{1-i}$ is a NO-instance, any potential solution should assign to *v* the value *i*. Hence we can safely delete from the constraints of $\mathcal {I}$ all the tuples setting *v* equal to $1-i$, and then remove variables and constraints that belong to ${\bar{c}} \cup \{v\}$.

### Corollary 12

Boolean CSP is fixed-parameter tractable w.r.t. the slim tree-cut width of the incidence graph.

Last but not least, given the ease with transferring dynamic programming algorithms from edge-cut width or degree treewidth to slim tree-cut width, an inquisitive reader might be wondering whether it is not possible to formally prove that *every* problem which is FPT w.r.t. former is also FPT w.r.t. the latter. That is, however, not true in general: one can construct entirely artificial problems which do not behave in this way.

To illustrate this on a high level for edge-cut width, let us consider an arbitrary graph problem $P$ which remains NP-hard even on trees (as an example, the Firefighter problem [12]) and can be solved on general *n*-vertex graphs in time $\tau (n)$. Moreover, let $\gamma (n)$ denote the time required to compute the slim tree-cut width of a graph *G* via an exhaustive brute force search, and let $\psi$ be a function which dominates both $\tau$ and $\gamma$. We now define an artificial new problem $P'$ as follows:

- every *n*-vertex graph *G* such that $\psi ({\text {ecw}}(G))\le n$ is a YES-instance, and otherwise
- *G* is a YES-instance if and only if *G* is a YES-instance of Firefighter.

Then $P'$ is FPT parameterized by edge-cut width. Indeed, given an instance (*G*, *k*) of $P'$, one can attempt to run a brute-force search to determine the edge-cut width (which is promised to be at most *k*) with a time-out of $\psi (\psi (k))$. If the algorithm times out, this implies that $\psi ({\text {ecw}}(G))\le n$ and we correctly output “Yes”. If not, we proceed by calling a brute-force algorithm to solve Firefighter on *G*, and this must once again complete in time at most $\psi (\psi (k))$. On the other hand, $P'$ remains NP-hard even on graph classes with constant ${\text {stcw}}(G)$—consider, for instance, the class of all graphs with two connected components, one of which is a tree and the other a graph from the class with constant slim tree-cut width but unbounded edge-cut width (one such class is depicted in Figure 2 of [4]). On some inputs from this class, $P'$ will ask for a solution to the Firefighter problem (which is NP-hard on trees) but the parameter ${\text {stcw}}(G)$ will remain constant.

## Conclusion

The contribution of this work is mainly conceptual: it provides a possible resolution to the search for an alternative to treewidth for edge cuts which is both structurally sound and exhibits the expected (and desired) algorithmic properties. Slim tree-cut width can be viewed as the “missing link” which explains why the problems depicted in Table 1 admit fixed-parameter algorithms that exploit dynamic programming along small edge cuts w.r.t. both edge-cut width (as a generalization of the feedback edge number) and treewidth plus maximum degree. We firmly believe that there are many more problems of interest where edge-cut based parameters may help push the frontiers of tractability. On this front, the alternative characterization via the edge-cut width of a supergraph provides decompositions which are better suited for dynamic programming than tree-cut decompositions.

The problem of computing optimal decompositions for slim tree-cut width remains, similarly as in the case of tree-cut width [28], as a prominent open question. Moreover, we believe that the ideas used to obtain a 2-approximation algorithm for tree-cut width could also be used to obtain an improved constant-factor approximation for slim tree-cut width.

## Footnotes

1 We view parameter $\alpha$ as being more restrictive than parameter $\beta$ if every graph class where $\alpha$ is bounded also has bounded $\beta$, but the opposite does not hold.

2 Formal definitions are provided in Sect. 2.
